# Impact of a large deletion in the neuraminidase protein identified in a laninamivir‐selected influenza A/Brisbane/10/2007 (H3N2) variant on viral fitness *in vitro* and in ferrets

**DOI:** 10.1111/irv.12356

**Published:** 2016-01-29

**Authors:** Julie Ann, Yacine Abed, Edith Beaulieu, Xavier Bouhy, Marie‐Hélène Joly, Karen Dubé, Julie Carbonneau, Marie‐Eve Hamelin, Corey Mallett, Guy Boivin

**Affiliations:** ^1^ CHU de Quebec Research Center (CHUL) and Laval UniversityQuébec CityQCCanada; ^2^ GSK VaccinesLavalQCCanada

**Keywords:** A(H3N2), deletion, influenza, neuraminidase, resistance

## Abstract

Viral fitness of a laninamivir‐selected influenza A/Brisbane/10/2007‐like (H3N2) isolate (LRVp9) containing a 237‐amino acid neuraminidase deletion and a P194L hemagglutinin mutation was evaluated *in vitro* and in ferrets. LRVp9 and the wild‐type (WT) virus showed comparable replication kinetics in MDCK‐ST6GalI cells. Cultured virus was recovered between days 2 and 5 post‐infection in nasal washes (NW) from the 4 WT‐infected ferrets whereas no virus was recovered from the LRVp9‐infected animals. There was a ≥1 log reduction in viral RNA copies/μl of NW for LRVp9 compared to WT at most time points. The large neuraminidase deletion compromises viral infectivity *in vivo*.

## Introduction

Influenza A(H3N2) viruses have been continuously circulating during seasonal influenza epidemics since 1968, and infections with these viruses have generally been associated with greater morbidity and mortality than A(H1N1) viruses.^1^ As most seasonal A(H3N2) viruses isolated after 2005 are resistant to adamantanes (amantadine and rimantadine),^2^ neuraminidase inhibitors (NAIs) constitute the antiviral option of choice against these infections. This class currently includes the globally used oseltamivir phosphate (Tamiflu, Hoffmann‐La Roche) and zanamivir (Relenza, GlaxoSmithKline),^3^ in addition to peramivir (Rapivab, BioCryst) whose parenteral formulation is available in Japan, South Korea, and the USA 4, 5 and the long‐acting NAI, laninamivir octanoate (Inavir, Daiichi Sankyo), which has been approved in Japan.^6^, ^7^, ^8^ As for other antivirals, resistance to NAIs in seasonal A(H3N2) viruses may constitute a serious clinical problem. Therefore, studies on the mechanisms of resistance to NAIs and characterization of fitness properties of NAI‐resistant A(H3N2) variants are of significant importance.

We recently used an influenza A/Brisbane/10/2007‐like (H3N2) virus for *in vitro* passages in cells overexpressing α2,6 receptors (ST6GalI‐MDCK) in presence of laninamivir.^9^ A selected variant obtained at the 9th passage in presence of laninamivir (LRVp9) had a large deletion (Del 106‐342) in the NA that was accompanied by a P194L (H3 numbering) mutation in the receptor binding site (RBS) of the hemagglutinin (HA). This genotype was associated with a loss of NA activity, and the virus growth was not inhibited by the presence of high concentrations of laninamivir and oseltamivir in the agar overlay during susceptibility assessment with plaque reduction assays.^9^ LRVp9 also had altered binding to turkey and guinea pig red blood cells and grew in ST6GalI‐MDCK cells both in presence or absence of laninamivir.^9^ As little information is available on the viral fitness of laninamivir‐resistant A(H3N2) viruses, the aim of this study was to evaluate the impact of laninamivir‐induced NA/HA changes on replicative capacity *in vitro* and infectivity in ferrets.

## Methods

The control‐passaged A/Brisbane/10/2007‐like wild‐type (WT) virus and LRVp9 were used to infect ST6GalI‐MDCK cells (kindly provided by Dr. Y. Kawaoka, Department of Pathological Sciences, School of Veterinary Medicine, University of Wisconsin, Madison) at a multiplicity of infection of 0·0001 PFU/cell. The infection medium consisted of Dulbecco's modified Eagle's medium (DMEM) containing 1 μg/ml of TPCK‐treated trypsin. Supernatants were collected every 12 h until 84 h post‐infection (p.i.) and titrated by plaque assays using ST6GalI‐MDCK cells.

Groups of 4 seronegative (900‐ to 1500‐g) male ferrets (Marshall BioResources, North Rose, NY) were housed in individual cages separated to prevent cage to cage transmission. Animals were lightly anesthetized by isoflurane and received an intranasal instillation of 4·5 log TCID50/ml in a total volume of 250 μL (125 μL per nostril) of the WT virus or the LRVp9 variant. Nasal wash (NW) samples were collected on a daily basis until day 10 p.i. in awake animals by instillation of 5 ml of PBS into the external nares of animals. To avoid repeated anesthesia during NW sampling that may have an impact on the ferrets welfare, animals were acclimatized to human handling during 10 days prior to viral inoculation. Viral titers of NW samples were determined by plaque assays in ST6GalI‐MDCK cells. A quantitative real‐time RT‐PCR targeting the influenza matrix (M) gene was performed for detection of viral RNA in NW.^10^ Sera were collected on days 0 and 14 p.i. to evaluate seroconversion by HA inhibition (HAI) assays.

### Ethics statement

Animal procedures were approved by the Institutional Animal Care Committee of Armand Frappier Institute according to the guidelines of the Canadian Council on Animal Care.

## Results

**Figure 1:**
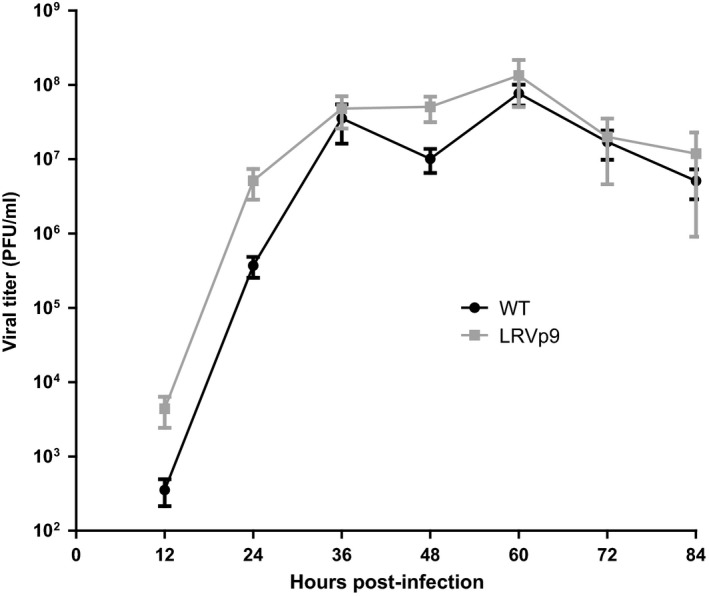
*In vitro* replicative capacities of influenza A/Brisbane/10/2007 (H3N2) WT virus and its LRVp9 variant. Viral titers were determined at the indicated time points from supernatants of ST6GalI‐MDCK cells infected at a multiplicity of infection (MOI) of 0·0001. Mean viral titers ± standard deviations from triplicate values of two independent experiments (*n* = 6) were determined using standard plaque assays.

The WT and LRVp9 viruses showed comparable replication kinetics *in vitro*. The peak titer for both viruses (≈ 10^8^ PFU/ml) was reached at 60 h p.i. (Figure 1). Sequencing of the viral HA/NA genes recovered at 72 h p.i. confirmed the genotypes present in the respective inoculum with no additional changes.

All ferrets were seronegative [HAI titers ≤20 for A/Uruguay/716/2007 (H3N2), an A/Brisbane/10/2007‐like strain] before viral inoculation. The 4 ferrets inoculated with the WT virus seroconverted with HAI titers of 640–1280 on day 14 p.i. whereas only one ferret from the LRVp9 group seroconverted (HAI titer of 160).

**Figure 2:**
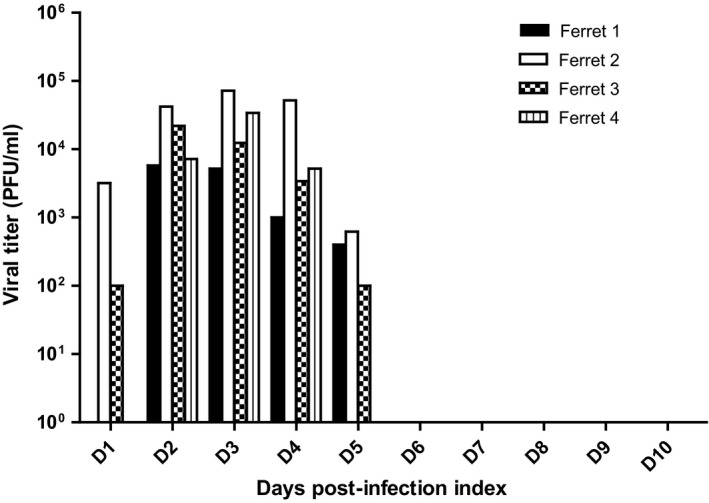
Nasal wash viral titers of ferrets infected with influenza A/Brisbane/10/2007 (H3N2) WT virus. Viral titers for each WT‐infected ferret were determined using standard plaque assays from nasal wash samples collected at the indicated days post‐inoculation. Note that no infectious virus was recovered from LRVp9‐infected ferrets.

**Figure 3:**
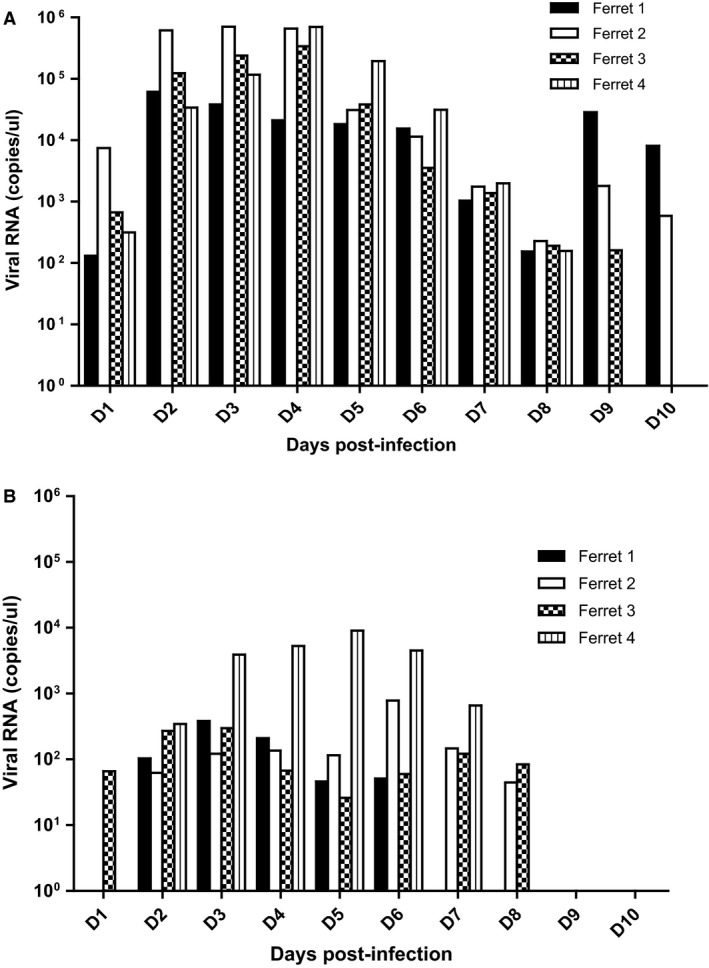
Quantification of viral RNA in nasal wash samples of ferrets infected with influenza A/Brisbane/10/2007 (H3N2) WT virus (A) and its LRVp9 variant (B). The number of viral RNA copies in nasal wash samples collected at the indicated days post‐inoculation was determined by real‐time RT‐PCR targeting the influenza matrix (M) gene.

Ferrets inoculated with the WT virus had a mean peak viral titer in NW samples of 3 **× **10^4^ PFU/ml on day 2/3 p.i. (range 5·2 **× **10^3^ to 7·2 **× **10^4^ PFU/ml). The ferrets cleared the virus by day 6 after infection (Figure 2). The ferrets inoculated with LRVp9 did not shed infectious virus. Viral RNA could be detected by quantitative RT‐PCR in NW of the four ferrets inoculated with the WT virus on day 1 p.i. (range 1·31 **× **10^2^ to 7·44 **× **10^3^ RNA copies/μl) with two animals (ferrets 1 and 2) remaining positive on day 10 p.i. (8·1 **× **10^3^ and 5·85 **× **10^2^ RNA copies/μl, respectively) (Figure 3A). By contrast, only one ferret from the mutant group was positive on day 1 p.i. (with 6·59 **× **10^1^ RNA copies/μl) while no viral RNA could be detected in any ferret after day 8 p.i. (Figure 3B). Although not statistically significant by unpaired *t*‐test, there was a ≥1 log reduction in viral RNA copies/μl for the mutant samples vs respective WT samples at most time points (Figure 3). Of note, viruses sequenced on day 6 p.i. displayed expected genotypes (Figure S1).

## Discussion

In this study, we evaluated the viral fitness of an NAI‐resistant influenza A/Brisbane/10/2007‐like (H3N2) strain containing a large NA deletion and a P194L HA substitution that emerged under *in vitro* laninamivir pressure. This variant corresponds to the 9th passage in which the growth medium contained 2 μM of laninamivir.^9^ In addition to the P194L mutation, the HA protein of this variant contained an S138A substitution, which is unlikely to be linked to the NAI pressure as it was also detected in the virus that was subjected to 9 passages without drug.^9^ When blast analyses using GenBank database sequences were performed, the 194L HA genotype was detected in numerous American, Asian, and Australian A(H3N2) viruses that have circulated since 2007 whereas no large NA deletion equivalent to that of LRVp9 could be detected. Residue 194 of influenza A(H3N2) HA is located at the globular head of the molecule and is part of the RBS. Therefore, changes at this position could naturally occur during influenza evolution contrasting to the internal region of NA protein which contains the highly conserved active site whose deletion seems to be strictly linked to the NAI pressure.

Previous studies already described the effect of NAI‐resistance framework 11 and functional 12 NA substitutions on infectivity of A(H3N2) variants using the ferret model. However, the impact of large NA deletions on viral fitness of A(H3N2) viruses remains poorly studied. Mishin and colleagues previously described the properties of A(H1N1) variants harboring a large NA deletion in ferrets.^13^ Nevertheless, the variant of that study did not contain any HA change. Thus, assessing the impact of the NA/HA changes identified in our A(H3N2) variant on viral fitness *in vitro* and in ferrets was particularly relevant. Despite efficient replication of the LRVp9 mutant in ST6GalI‐MDCK cells, no cytopathic effects could be seen when we used native MDCK cells. Evidence of altered binding to red blood cells of LRVp9 was previously demonstrated using HA‐elution assays.^9^ On the other hand, viral infectivity of LRVp9 was severely impaired in ferrets. This could be explained by the absence of NA activity as the catalytic head domain containing the active site of the NA enzyme was missing. Interestingly, previous reports demonstrated that the functional R292K NA substitution, which causes a significant reduction of NA activity, was associated with decreased infectivity and transmissibility of NAI‐resistant A(H3N2) viruses in ferrets.^11^ Therefore, a certain level of NA activity seems to be necessary for efficient infectivity and transmissibility of A(H3N2) viruses *in vivo*.

A functional balance between the NA and HA of influenza viruses is necessary for both efficient replication and transmission of influenza viruses.^14^ Thus, NA mutations that reduce the NA activity could be compensated by HA mutations that reduce viral binding. This could be the case of the P194L mutation within the RBS of the HA glycoprotein. Notably, the P194L HA substitution was also selected together with the E119A NA substitution in a NAI‐resistant influenza A(H5N1) variant under oseltamivir pressure.^15^

The infectivity of LRVp9 was impaired *in vivo*, with only 1 of 4 ferrets seroconverting, and this ferret did not shed any infectious virus. Contrasting with *in vitro* observations, the P194L HA mutation did not restore the viral fitness *in vivo*. Also, the absence of viral shedding in the ferrets inoculated with the mutant may be explained by the large NA deletion potentially impairing the liberation of new virions and their spread. The number of infectious particles in respiratory tract secretions is a determining factor for transmission by the airborne route or by direct contact.^12^ In that regard, the LRVp9 mutant is unlikely to be transmissible *in vivo*.

A limitation of our study is that we did not investigate the specific role of the NA and HA mutations that occurred in our drug‐selected A(H3N2) variant.

## Conclusions

In conclusion, we showed that the NA‐defective, HA‐mutated A(H3N2) variant conserved its viral fitness *in vitro,* but did not replicate in the upper respiratory tract of ferrets. Constant surveillance of the emergence of drug‐resistant virus variants with adequate evaluation of key biological properties in relevant animal models is necessary.

## Conflict of interests

GB has received research funds from Biota Scientific Management, GlaxoSmithKline Biologicals S.A., Hoffmann La Roche, and Abbott. EB, MHJ, KD, and CM are employees of the GSK group of companies.

## Supporting information


**Figure S1.** Multiple sequence alignment of the neuraminidase (panel A) or hemagglutinin (panel B) protein of the influenza A/Brisbane/10/07‐like H3N2 viruses used in this study was performed using clustal W (1.83).
