# Supplementary material for: Impact of a large deletion in the neuraminidase protein identified in a laninamivir‐selected influenza A/Brisbane/10/2007 (H3N2) variant on viral fitness in vitro and in ferrets
Source: Influenza Other Respir Viruses. 2016 Jan 29;10(2):122–6. doi: 10.1111/irv.12356 (PMC4746560; doi:10.1111/irv.12356)
Supplement: Supplementary file 1 — Figure S1. Multiple sequence alignment of the neuraminidase (panel A) or hemagglutinin (panel B) protein of the influenza A/Brisbane/10/07‐like H3N2 viruses used in this study was performed using clustal W (1.83). [file IRV-10-122-s001.docx]

**Panel A**

CP9 MNPNQKIITIGSVSLTISTICFFMQIAILITTVTLHFKQYEFNSPPNNQVMLCEPTIIER

NW-CP9 MNPNQKIITIGSVSLTISTICFFMQIAILITTVTLHFKQYEFNSPPNNQVMLCEPTIIER

LRVp9 MNPNQKIITIGSVSLTISTICFFMQIAILITTVTLHFKQYEFNSPPNNQVMLCEPTIIER

NW-LRVp9 MNPNQKIITIGSVSLTISTICFFMQIAILITTVTLHFKQYEFNSPPNNQVMLCEPTIIER

************************************************************

CP9 NITEIVYLTNTTIEKEICPKLAEYRNWSKPQCDITGFAPFSKDNSIRLSAGGDIWVTREP

NW-CP9 NITEIVYLTNTTIEKEICPKLAEYRNWSKPQCDITGFAPFSKDNSIRLSAGGDIWVTREP

LRVp9 NITEIVYLTNTTIEKEICPKLAEYRNWSKPQCDITGFAPFSKDN----------------

NW-LRVp9 NITEIVYLTNTTIEKEICPKLAEYRNWSKPQCDITGFAPFSKDN----------------

********************************************

CP9 YVSCDPDKCYQFALGQGTTLNNVHSNDTVRDRTPYRTLLMNELGVPFHLGTKQVCIAWSS

NW-CP9 YVSCDPDKCYQFALGQGTTLNNVHSNDTVRDRTPYRTLLMNELGVPFHLGTKQVCIAWSS

LRVp9 ------------------------------------------------------------

NW-LRVp9 ------------------------------------------------------------

CP9 SSCHDGKAWLHVCITGDDKNATASFIYNGRLVDSIVSWSKEILRTQESECVCINGTCTVV

NW-CP9 SSCHDGKAWLHVCITGDDKNATASFIYNGRLVDSIVSWSKEILRTQESECVCINGTCTVV

LRVp9 ------------------------------------------------------------

NW-LRVp9 ------------------------------------------------------------

CP9 MTDGSASGKADTKILFIEEGKIVHTSTLSGSAQHVEECSCYPRYPGVRCVCRDNWKGSNR

NW-CP9 MTDGSASGKADTKILFIEEGKIVHTSTLSGSAQHVEECSCYPRYPGVRCVCRDNWKGSNR

LRVp9 ------------------------------------------------------------

NW-LRVp9 ------------------------------------------------------------

CP9 PIVDINIKDHSTVSSYVCSGLVGDTPRKNDSSSSSHCLDPNNEEGGHGVKGWAFDDGNDV

NW-CP9 PIVDINIKDHSTVSSYVCSGLVGDTPRKNDSSSSSHCLDPNNEEGGHGVKGWAFDDGNDV

LRVp9 -----------------------------------------SEEGGHGVKGWAFDDGNDV

NW-LRVp9 -----------------------------------------SEEGGHGVKGWAFDDGNDV

.******************

CP9 WMGRTISEKSRLGYETFKVIEGWSNPKSKLQINRQVIVDRGNRSGYSGIFSVEGKSCINR

NW-CP9 WMGRTISEKSRLGYETFKVIEGWSNPKSKLQINRQVIVDRGNRSGYSGIFSVEGKSCINR

LRVp9 WMGRTISEKSRLGYETFKVIEGWSNPKSKLQINRQVIVDRGNRSGYSGIFSVEGKSCINR

NW-LRVp9 WMGRTISEKSRLGYETFKVIEGWSNPKSKLQINRQVIVDRGNRSGYSGIFSVEGKSCINR

************************************************************

CP9 CFYVELIRGRKEETEVLWTSNSIVVFCGTSGTYGTGSWPDGADINLMPI

NW-CP9 CFYVELIRGRKEETEVLWTSNSIVVFCGTSGTYGTGSWPDGADINLMPI

LRVp9 CFYVELIRGRKEETEVLWTSNSIVVFCGTSGTYGTGSWPDGADINLMPI

NW-LRVp9 CFYVELIRGRKEETEVLWTSNSIVVFCGTSGTYGTGSWPDGADINLMPI

*************************************************

**Panel B**

CP9 MKTIIALSYILCLVFTQKLPGNDDSTATLCLGHHAVPNGTIVKTITNDQIEVTNATELVQ

NW-CP9 MKTIIALSYILCLVFTQKLPGNDDSTATLCLGHHAVPNGTIVKTITNDQIEVTNATELVQ

LRVp9 MKTIIALSYILCLVFTQKLPGNDDSTATLCLGHHAVPNGTIVKTITNDQIEVTNATELVQ

NW-LRVp9 MKTIIALSYILCLVFTQKLPGNDDSTATLCLGHHAVPNGTIVKTITNDQIEVTNATELVQ

************************************************************

CP9 SSSTGEICDSPHQILDGENCTLIDALLGDPQCDGFQNKKWDLFVERSKAYSNCYPYDVPD

NW-CP9 SSSTGEICDSPHQILDGENCTLIDALLGDPQCDGFQNKKWDLFVERSKAYSNCYPYDVPD

LRVp9 SSSTGEICDSPHQILDGENCTLIDALLGDPQCDGFQNKKWDLFVERSKAYSNCYPYDVPD

NW-LRVp9 SSSTGEICDSPHQILDGENCTLIDALLGDPQCDGFQNKKWDLFVERSKAYSNCYPYDVPD

************************************************************

CP9 YASLRSLVASSGTLEFNNESFNWTGVTQNGTSSACIRRS**K**NSFFSRLNWLTHLKFKYPAL

NW-CP9 YASLRSLVASSGTLEFNNESFNWTGVTQNGTSSACIRRS**K**NSFFSRLNWLTHLKFKYPAL

LRVp9 YASLRSLVASSGTLEFNNESFNWTGVTQNGTSSACIRRS**N**NSFFSRLNWLTHLKFKYPAL

NW-LRVp9 YASLRSLVASSGTLEFNNESFNWTGVTQNGTSSACIRRS**N**NSFFSRLNWLTHLKFKYPAL

***************************************:********************

CP9 NVTMPNNEKFDKLYIWGVHHPGTDNDQIF**P**YAQASGRITVSTKRSQQTVIPNIGSRPRVR

NW-CP9 NVTMPNNEKFDKLYIWGVHHPGTDNDQIF**P**YAQASGRITVSTKRSQQTVIPNIGSRPRVR

LRVp9 NVTMPNNEKFDKLYIWGVHHPGTDNDQIF**L**YAQASGRITVSTKRSQQTVIPNIGSRPRVR

NW-LRVp9 NVTMPNNEKFDKLYIWGVHHPGTDNDQIF**L**YAQASGRITVSTKRSQQTVIPNIGSRPRVR

***************************** ******************************

CP9 NIPSRISIYWTIVKPGDILLINSTGNLIAPRGYFKIRSGKSSIMRSDAPIGKCNSECITP

NW-CP9 NIPSRISIYWTIVKPGDILLINSTGNLIAPRGYFKIRSGKSSIMRSDAPIGKCNSECITP

LRVp9 NIPSRISIYWTIVKPGDILLINSTGNLIAPRGYFKIRSGKSSIMRSDAPIGKCNSECITP

NW-LRVp9 NIPSRISIYWTIVKPGDILLINSTGNLIAPRGYFKIRSGKSSIMRSDAPIGKCNSECITP

************************************************************

CP9 NGSIPNDKPFQNVNRITYGACPRYVKQNTLKLATGMRNVPEKQTRGIFGAIAGFIENGWE

NW-CP9 NGSIPNDKPFQNVNRITYGACPRYVKQNTLKLATGMRNVPEKQTRGIFGAIAGFIENGWE

LRVp9 NGSIPNDKPFQNVNRITYGACPRYVKQNTLKLATGMRNVPEKQTRGIFGAIAGFIENGWE

NW-LRVp9 NGSIPNDKPFQNVNRITYGACPRYVKQNTLKLATGMRNVPEKQTRGIFGAIAGFIENGWE

************************************************************

CP9 GMVDGWYGFRHQNSEGIGQAADLKSTQAAIDQINGKLNRLIGKTNEKFHQIEKEFSEVEG

NW-CP9 GMVDGWYGFRHQNSEGIGQAADLKSTQAAIDQINGKLNRLIGKTNEKFHQIEKEFSEVEG

LRVp9 GMVDGWYGFRHQNSEGIGQAADLKSTQAAIDQINGKLNRLIGKTNEKFHQIEKEFSEVEG

NW-LRVp9 GMVDGWYGFRHQNSEGIGQAADLKSTQAAIDQINGKLNRLIGKTNEKFHQIEKEFSEVEG

************************************************************

CP9 RIQDLEKYVEDTKIDLWSYNAELLVALENQHTIDLTDSEMNKLFEKTKKQLRENAEDMGN

NW-CP9 RIQDLEKYVEDTKIDLWSYNAELLVALENQHTIDLTDSEMNKLFEKTKKQLRENAEDMGN

LRVp9 RIQDLEKYVEDTKIDLWSYNAELLVALENQHTIDLTDSEMNKLFEKTKKQLRENAEDMGN

NW-LRVp9 RIQDLEKYVEDTKIDLWSYNAELLVALENQHTIDLTDSEMNKLFEKTKKQLRENAEDMGN

************************************************************

CP9 GCFKIYHKCDNACIGSIRNGTYDHEVYRDEALNNRFQIKGVELKSGYKDWILWISFAISC

NW-CP9 GCFKIYHKCDNACIGSIRNGTYDHEVYRDEALNNRFQIKGVELKSGYKDWILWISFAISC

LRVp9 SCFKIYHKCDNACIGSIRNGTYDHEVYRDEALNNRFQIKGVELKSGYKDWILWISFAISC

NW-LRVp9 SCFKIYHKCDNACIGSIRNGTYDHEVYRDEALNNRFQIKGVELKSGYKDWILWISFAISC

.***********************************************************

CP9 FLLCVALLGFIMWACQKGNIRCNICI

NW-CP9 FLLCVALLGFIMWACQKGNIRCNICI

LRVp9 FLLCVALLGFIMWACQKGNIRCNICI

NW-LRVp9 FLLCVALLGFIMWACQKGNIRCNICI

**************************
